# Immunity and the coral crisis

**DOI:** 10.1038/s42003-018-0097-4

**Published:** 2018-07-09

**Authors:** Caroline V. Palmer

**Affiliations:** 1 School of Biological & Marine Sciences, University of Plymouth, Plymouth, Devon PL48AA UK; 2 Guanacaste Dry Forest Conservation Fund, Devon, PL207NA UK

## Abstract

Climate change is killing coral at an unprecedented rate. As immune systems promote homeostasis and survival of adverse conditions I propose we explore coral health in the context of holobiont immunity.

## Abstract (web summary)

Caroline Palmer proposes the concept of coral holobiont damage thresholds to stimulate research into coral health and immunity as tropical reefs are increasingly threatened by climate change. This framework may be used to develop targeted approaches to coral reef restoration, management and conservation.

## Immunity, purveyor of health

Immune systems are the purveyors of homeostasis and orchestrators of relationships among hosts, mutualists, commensals and pathogens of multipartite organisms, termed holobionts. Immunity underpins the health and survival of these holobionts—withstanding disruption and re-establishing homeostasis in the face of biotic and abiotic perturbations. Physiological trade-offs operating under evolved constraints drive distinct manifestations of immunity and underpin resistance. Variation in immunity therefore likely has a strong influence over coral survivorship under climate-change pressures.

Traditionally, innate immunity was recognized as the system used by invertebrates as a non-specific response to non-self^1^. But non-specificity and non-self may be out-dated concepts; innate immune systems have targeted responses and memory^2,3^, and many organisms are not discrete ‘self’ entities with borders to defend^4^. Corals are complex mutualisms among multicellular partners and associated microbiota, which influence coral physiology *sensu latu*^5,6^. Corals and their plethora of mutualists, including the light-harvesting and energy-providing algae *Symbiodinium* spp. that live inside coral cells, enable all associates to thrive in often nutrient-poor tropical warm waters and build complex reef structures^7^. Homeostasis of these coral holobionts^8^ therefore hinges on the immune system accepting mutualists and being vigilant of imposters while managing the housekeeping: clearing dead cells and selecting and maintaining an appropriate commensal microbiota^9^.

With climate change impacting the entire global marine ecosystem, as well as millions of people, biologists are racing to develop novel approaches to better conserve, restore and manage coral reefs^10^, which have endured significant impacts since the early 1980s^11^. To be effective, we need to fully explore, and embrace, the intricacies and complexities of coral holobiont health^12^. This will require understanding the role of immunity in maintaining and degrading homeostasis, inclusive of mutualisms and under environmental change^13,14^.

Understanding the dynamics and limitations of coral holobiont immunity is essential for accurately interpreting stress experiments and for elucidating appropriate target genes for assisted evolution of more resilient corals. To stimulate research, I challenge the dogma of coral bleaching as a general stress response distinct from immunity. I also propose the Damage Threshold Hypothesis of Coral Holobiont Susceptibility, a related concept to that described previously for insects^15^, to conceptualize immune-dynamics under homeostasis and with perturbations.

## Homeostasis of self

Immunity is generally treated as an activated response—being switched on from an off state^16^ during an invasion. However, heightened immune activity is just one of various modalities in which an immune system operates. Immune systems can be viewed as ecosystems of receptors, cells, pathways, chemical mediators, mutualists, invaders, and more. These immune ecosystems therefore maintain homeostasis of health by being constantly active, assessing, checking and making adjustments through continual, normal, immune activity—termed constituent immunity—like our circulating white blood cells and the turn-over of our skin cells^16,17^. A lack of heightened immune activity in the presence of normally-associated microbiota indicates a tolerance of them, rather than an inactive immune system. This normal conglomerate of organisms, the coral holobiont^8^, arguably delimits immune self^16^.

The coral holobiont immune self can be viewed as comparable to the vertebrate or plant with its mitochondria and plastids, though at a much earlier stage of co-evolution. Similar to plants, coevolution of the coral holobiont has occurred under inescapable daily and seasonal fluctuations in abiotic conditions. For plants, this sedentary existence has ensured the necessary development of a finely tuned, integrated and responsive system for maintaining health in the presence of multiple stresses, both biotic and abiotic^18^. Similarly, the coral holobiont, like those of other organisms^19,20^, is likely to be maintained in dynamic equilibrium by an interconnected immune system that promotes health and protects partnerships for optimal function, moderates commensals, removes over-abundant, degraded or malfunctioning mutualists^21^ and embraces new ones^14,22^ when circumstances warrant. However, the limits of coral holobiont immune systems are being tested as accelerating climate change shifts baseline abiotic conditions and fosters extremes.

## The dynamics of immunity

The manifestation of an immune system—including both constituent immunity and immune responses—is variable among and within organisms. This is largely because it requires resources that may otherwise go to other functions^23^ at both ecological and evolutionary scales^24^. In this respect, at any given moment, an organism’s immune strategy is the consequence of circumstance-dependent, and phenotypically plastic, physiological trade-offs, occurring within the predetermined constraints of evolutionary trade-offs, as per Life History Theory^23^. Physiological costs, determining plasticity, can be incurred during both the maintenance and implementation of immunity, and include energy expenditure and potential self-harm (autoimmunity)^24^. For many organisms, including coral^25^, such physiological costs may be expressed as a temporary reduction in fecundity or growth, as resources are reallocated, and/or damaged by the immune response^24^. Coral holobiont homeostasis, as maintained by immunity, is therefore likely directly related to energy availability and the ability to compensate for costs—such as through increased heterotrophy. For organisms, likely including corals, the varying timescales and difficult-to-measure variables, plus potentially hidden compensations^24^, make the costs of immunity difficult to quantify^26^. However, the variability in cost allocation is likely responsible for the great variation in both constituent immunity and immune responses observed among and within corals^13,27^.

## Danger model challenges the coral general stress response dogma

Since coral bleaching—the breakdown in mutualism between coral holobiont and *Symbiodinium*—was first described^28^, it has largely become synonymous with generic terms including general stress response, stress response or thermal stress response^29–32^. Used in reference to coral responses ranging in scale from the gene^33^ to ecosystem^32,34^, such terms likely came into common use because of the plethora of stressors that induce coral bleaching^29,35^. Coral bleaching, and the assumed general stress response, has been a dominant focus of coral health biology for decades, due to its high impact on reefs globally^29,33,36–39^. Easy to use and encompassing a wide variety of biological contexts, meanings and mechanisms, coral ‘stress responses’ are often investigated and discussed with little to no reference to immunity, or as a completely separate biological process^33,39,40^. The continued segregation of stress responses and immunity in the coral literature^33,40^, or omission of immunity in reference to resilience^6,10^, has the effect that they are generally treated as distinct. In reality, stress responses and immunity are intrinsically linked and, particularly in the context of the holobiont, are more likely one and the same^14,21,41^.

In general biology, a stress response typically refers to a hormone-mediated fight or flight reaction^42^, but can also refer to the integrated stress response (ISR)—a conserved, context-dependent cellular pathway that promotes survival^43^. ISR, or cellular stress response, is often characterized by a pathogen-killing oxidative burst^44^ that can lead to damaging oxidative stress^45^. Since oxidative stress has been reported as a driver of coral-algal mutualism breakdown^14,35,46^, components of the ISR pathway, including reactive species (e.g., nitric oxide), and antioxidants have been extensively detected in coral holobionts as stress responses to adverse conditions (such as warmer water^30,47,48^) and with infection^31^. Consequently, components of the ISR pathway have been proposed as potential gene expression biomarkers (GEB) of thermal stress in the coral holobiont^40^. However, such so-called stress components may be described as immune modulators^49^.

Immune responses are triggered upon the detection of danger and/or by a disruption in the structure or function of cells, tissues or mutualisms, to a level that perturbs holobiont homeostasis and affects fitness. Disruptors of homeostasis and elicitors of immunity, may be biotic, abiotic or a combination of both^20^. They may also be exogenous, such as pathogenic infection, or endogenous such as tumorous growth or cellular instability^50^. The mechanism of immune signaling to abiotic stress is conserved from plants^51^ to humans^49^, and occurs with the extracellular release or leakage of endogenous cell constituents or components^45^. Such components are considered key to stress responses of cells^45^. In the Danger Model of immune activity^49^ cellular ‘danger’ molecules, including heat shock proteins, uric acid and reactive molecules, signal damage and are essential in modulating an effective immune response to various perturbations^49,51,52^. The coral general stress response, and resultant bleaching, is therefore a component of holobiont immunity^14,21^. The continued use of terms such as ‘general stress response’, in the absence of reference to immune activity will likely continue to polarize coral health research and handicap our understanding of the acclimation and adaptation potential of corals—something that is being actively investigated^53,54^.

## Combining Self/non-self and Danger theories of immunity

**Fig. 1:**
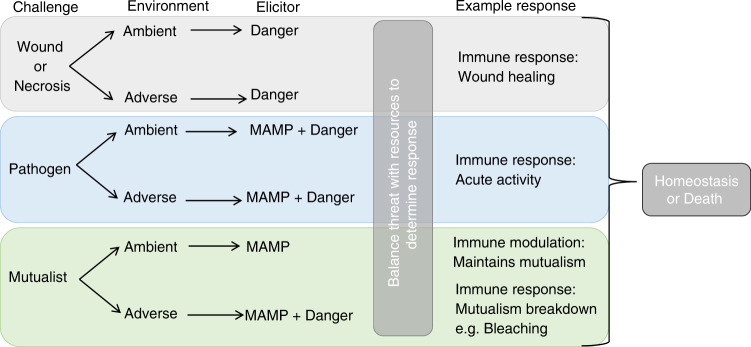
MAMP detection, danger signaling and available resources determine immune response to disruption. The total disruption of homeostasis is the combination of a challenge with environmental conditions, which can be detected as danger, MAMPs or both. The elicitors detected, including mutualist-derived danger signals, and the available resources determine the type and magnitude of the immune response, which is also traded off against other factors including potential autoimmunity^20^

Self/non-self theory, involving suites of pattern recognition receptors that recognize conserved pathogens motifs, was largely accepted to explain pathogen detection and the induction of immune responses^55,56^. Corals possess many pattern recognition receptors, such as Toll-like receptors, Toll/interleukin-1 receptors and Lectins, which are able to detect microbe-associated molecular patters (MAMPs) and activate signaling pathways so as to produce immune responses (reviewed in Mydlarz, et al.^41^). However, MAMP detection with subsequent microbe killing by the holobiont immune system confounds the presence of specific mutualist and commensal interactions^20^. Equally conflicting is that immune responses may occur in the absence of a pathogen, such as with sterile wounding^49,57^, and disruption may not occur in the presence of non-self (e.g., coral chimeras^58^). These discrepancies, as well as the extensive crosstalk among pathways triggered by both biotic and abiotic perturbations within coral^33,41,59–61^, plants^18,62^ and vertebrates alike^63^, support the suggestion that Self/Non-self Theory alone does not fully represent the dynamics of immune activity. Combining two theories of immune activation, Self/Non-self Theory and the Danger Hypothesis, may hold the key to understanding coral holobiont immune dynamics (Fig. 1)^20,64^.

Coral immune activity, like that of insects^20^ and similar to that of plants^18^ and vertebrates^65^, is likely a balanced consequence of both MAMP^1,56^ and danger/damage signal (DAMP—danger associated molecular patterns) detection by pattern recognition receptors^49,66^ (Fig. 1). Depending on the combination of signals, their intensity and the current condition of the holobiont, an immune response may or may not be induced (Fig. 1)^20^. Viewing the coral holobiont immune system in this regard gives rise to the potential for context-dependent immune responses and microbiome modulation, which in turn addresses how corals are able to fight pathogens while maintaining their microbiota^67^. For example, under homeostatic conditions, commensal and mutualistic microbes are detected through pattern recognition receptors of MAMPs, but, in the absence of a danger signal (DAMP), do not elicit an immune response to eliminate them^20^ (Fig. 1). Furthermore, as a change in abiotic factors can induce the release of danger molecules from mutualists^45^, including coral endo-mutualists *Symbiodinium* spp.^14,48,68,69^, this combination of theories poses a mechanism by which mutualists, and even commensals, may be detected as foe (Fig. 1). However, the immune response elicited may be directly dependent on the physiological context, such as energetic reserves needed to mount an effective response and compensate for autoimmune damage. As such, we can overlay genetic and plastic variation in susceptibility to threats (e.g., disease and adverse environmental conditions^13,70^), and the observations that different holobionts are able to tolerate or resist a variable amount of challenge to homeostasis, dependant on current physiological constraints^24^.

## Damage threshold hypothesis of coral holobiont susceptibility

**Fig. 2:**
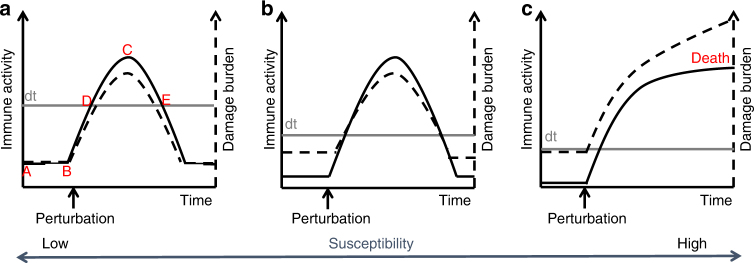
Damage threshold hypothesis of coral susceptibility. Holobiont with a low susceptibility, tolerance strategy (**a**) has comparatively high constituent immunity, (**b**) is intermediate, living closer to the lowered damage threshold (dt) and (**c**) respresents a highly susceptible holobiont, unable to survive the hypothetical perturbation. **a** indicates constituent immunity levels, while A and B demonstrate homeostatic tolerance of damage, lowering the burden. C and D represent the magnitude of heightened immunity to a perturbation and D and E indicate resilliance (the time taken to return below the damage threshold) with the shorter duration indicating higher resilience

Immune systems can use resistance or tolerance strategies to promote fitness in the presence of a perturbation^71–74^ (Box 1). The damage threshold hypothesis of insect-pathogen interactions^15^ proposes that resistance and tolerance are intimately related to the amount of damage/danger signaling an infection incurs, and its effect on fitness. As such, we can expand upon the damage threshold hypothesis^15^ to form a hypothetical and dynamic framework for how coral holobionts may moderate mutualisms, cohabit with commensals, kill pathogens and manage acute abiotic perturbations (Box 1). While the terms tolerance and resistance, and even resilience are often used interchangeably in coral biology^53,54^, they represent different immune strategies and outcomes^72^. Resisting a perturbation incurs high short-term costs, energetically, in rapidly mounting a strong immune response, and in autoimmune damage^24^. Tolerance, on the other hand, incurs a lower but longer-term cost of continual immune activity that physiologically offsets a damage burden, such as oxidative stress, and possibly more strictly moderates the microbiome^15^. The damage threshold hypothesis of coral holobiont susceptibility (Box 1, Fig. 2) demonstrates how more tolerant holobionts, with their higher constituent immunity^13^, may be better able to maintain homeostasis through perturbations (inclusive of a functional microbiome), than holobionts that employ a resistance strategy, which may be more readily overwhelmed. Balancing the trade-offs in immune strategy (Box 1), in the context of life history theory and physiology, may help explain the high variation in susceptibility to perturbations observed among coral holobionts with their dynamic microbiota^13,67,75^ (Box 1).

### Box 1: Damage threshold hypothesis of coral holobiont susceptibility

The damage threshold hypothesis proposes a model for which a holobiont can coexist with a specific microbiota (tolerance) and remain vigilant to disturbance (resistance). How this is achieved likely varies among holobionts and is dependent upon evolved life history strategy and physiological trade-offs^24^, which combine to determine damage thresholds (Fig. 2). Damage thresholds, defined as the upper limit of damage that can be tolerated without causing harm, are inversely related to susceptibility.

Coral constituent immunity during homeostasis is directly, and inversely, related to disease and bleaching susceptibility^13^. Holobiont constituent immunity—potentially including the benefits conferred by mutualists—can physiologically off-set damage incurred by fluctuations in local or holobiont conditions, and therefore can be used as a proxy for determining damage thresholds. It can be hypothesized that high investment into constituent immunity equates to a high damage threshold and reduced damage burden, and therefore a low susceptibility, high tolerance immune strategy e.g., *Porites* spp. (Fig. 2a). When a severe perturbation occurs, inducing damage that exceeds the damage threshold, such as with a virulent pathogen, wounding, an acute shift in environmental conditions or dysfunctional mutualists, an immune response is triggered to resist and eliminate the threat, to repair the damage and re-establish homeostasis. For the tolerant strategist, with high constituent immunity, there is a large buffer before damage threshold is breached, suggesting that signs of stress, disease and bleaching will likely be delayed and less severe. Similarly, the immune response will likely be short lived, returning homeostasis rapidly (high resilience) and with lower up-regulation as compared to less tolerant strategists (e.g., Fig. 2b).

For the hypothetical intermediate strategist (Fig. 2b), constituent immunity enables a reasonably high damage threshold, though the damage burden during homeostasis is notably closer to it. This ensures that, at the onset of a perturbation, a higher magnitude response is required, and return to homeostasis (immune activity returning below the damage threshold) takes longer, demonstrating lower resilience. Holobionts with least investment into constituent immunity live the closest to the damage threshold (Fig. 2c) e.g., *Acropora* spp., and therefore are at highest risk of mortality in the face of a perturbation. Low constituent immunity means that a comparatively higher up-regulation is required, risking autoimmunity and a high short-term energetic cost—the resistance strategist. It may also mean that the holobiont is overcome before physiological measures can compensate for damage, and resistance measures may be overwhelmed, leading to death. While Fig. 2a–c can represent different holobionts with differing life histories, they may also represent one holobiont sliding from low to high susceptibility during a chronic perturbation.

## Coral bleaching and breaching the damage threshold

Under homeostatic conditions, the removal of unwanted algal mutualists from coral tissue is a normal and inconspicuous component of housekeeping by the immune system^35^. However, more commonly the term coral bleaching is used to refer to an observable paling of part or all of a coral, resulting from the loss of photosynthetic pigments and/or associated *Symbiodinium* spp.^29,35^. Coral bleaching, though widely reported as a general stress response, can perhaps more accurately be described as a visually striking immune response to disruption in holobiont homeostasis—one that has exceeded the damage threshold^15^ (Box 1).

Accelerating global climate change is more frequently pushing tropical water temperatures, and photosynthetically active radiation (PAR) intensities, to and beyond the upper thermal limits of coral holobionts^29^. The resultant mass coral bleaching events, with their high coral mortality, pose the greatest threat to global coral reef persistence^76^. Occurring since the 1980s, mass bleaching events are the consequence of many coral holobiont immune systems becoming overwhelmed—pushed, often irreversibly, beyond their damage thresholds—by increasingly extreme environmental conditions. However, there is high variation in coral holobiont susceptibility to such events. Some corals bleach quickly and may die, whereas others take a long time to bleach or may not bleach at all. Such variation in immune tolerance to abiotic stress may be explained using the damage threshold hypothesis of coral holobiont susceptibility (Box 2).

**Fig. 3:**
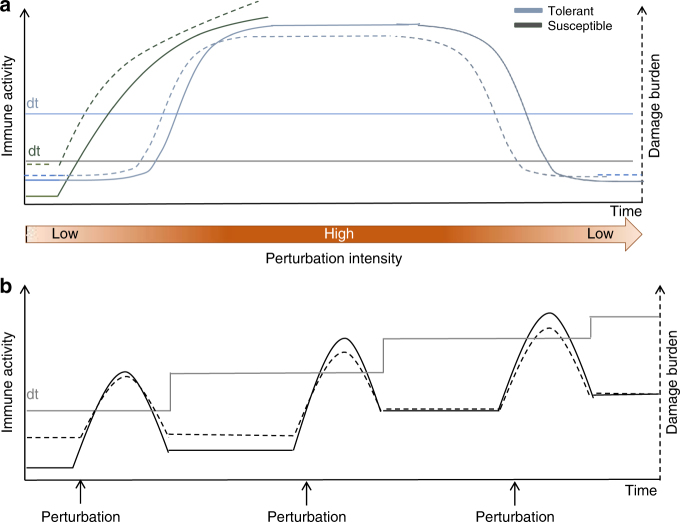
Damage threshold hypothesis (**a**) during a thermal bleaching event comparing how a tolerant and a suscpetible holobiont may respond, and (**b**) a potential mechanism for immune memory through mulitple perturbations

Thermal bleaching events are a relatively recent phenomenon that corals have not necessarily evolved with, and therefore present an unusual perturbation to holobiont homeostasis. Innate immune systems evolved to mitigate acute, short-lived events, such as an infection, with an equally acute immune response, though under daily and seasonal environmental fluctuations. The high short-term investment in immunity is justified by the rapid removal of the threat and limited, or localized, autoimmunity, which can be easily repaired or compensated for once the immediate threat is quelled. A long-term perturbation, such as sustained elevated water temperatures, presents a greater challenge. Holobionts with higher damage thresholds, higher constituent immunity, large energy reserves and/or the ability to compensate for depletions and autoimmune damage (e.g., by feeding and antioxidants), are better able to survive long-term perturbations like bleaching events (Box 2, Fig. 3)^30^. This raises the question of whether coral holobionts can be trained or modified to increase their damage thresholds and become better able to survive thermal extremes^6,77^.

### Box 2: Damage threshold hypothesis of coral holobiont susceptibility with persistent and recurring perturbations

Overlaying the damage threshold hypothesis of a tolerant and susceptible holobiont (Fig. 3a), we can begin to see how their immune responses may differ during a perturbation and why we might be misinterpreting results of stress experiments. Coral holobionts more susceptible to perturbations, such as those with low constituent immunity that invest preferentially into growth and/or reproduction (e.g., *Acropora* spp. and *Pocillopora* spp.) live closer to their damage thresholds. Therefore, damage thresholds are breached at a lower damage burden—earlier in a perturbation such as a thermal bleaching event—and heightened immune activity occurs earlier than for those living well below their damage thresholds (e.g., *Porites* spp.). This means that a more susceptible holobiont may initially demonstrate higher immune activity, including the breakdown of mutualisms (i.e., bleaching), to a perturbation than a more tolerant holobiont. However, this rapid and high-magnitude response may represent a flash-in-the-pan scenario, lowering energy stores and inducing a high autoimmune cost, and may ultimately be fatal. In this scenario, an unabating trigger such as a thermal anomaly, will continue to induce an immune response, but physiological mechanisms, such as antioxidants, are unable to compensate for the collateral damage. This likely leads to death, and explains why a greater immune response can lead to mortality in susceptible species^81^. The longer the perturbation, the more holobionts will reach an energetically depleted state where physiological mechanisms of even tolerant holobionts can no longer compensate for damage. This is likely what occurs during mass bleaching events.

However, the survivors of a perturbation may demonstrate immune memory^78^ and be more able to tolerate future perturbations^6,77^. One way in which this may occur is for constituent immunity to remain higher than before the perturbation e.g., Pinzon et al.^82^, thus raising the damage threshold over time (Fig. 3b).

## Immune memory, acclimatization and adaptation

Immune memory can be broadly defined as increased resistance upon re-exposure and was considered a phenomenon solely of adaptive immunity. However, innate immune systems of organisms including plants and invertebrates also, arguably, demonstrate immune memory, which is sometimes called priming^3^. In contradiction of innate immune systems being non-specific, typically, immune memory is investigated as a primed response to a specific parasitic infection, which subsides between challenges and provides resistance upon re-infection. Immune memory can also be an acquired resistance or sustained response that protects against a future challenge—an anticipatory response^3,78^. In addition to immune memory, innate immune systems, such as those of plants, display cross-tolerance, whereby responding to one perturbation increases tolerance or resistance to others^18^. Considering the coral holobiont immune system responds to both biotic and abiotic stimuli, immune memory with its heritability described for other invertebrates^3^, is a tantalizing topic in this era of climate change and coral reef crisis^79^.

Mechanisms of immune memory vary among organisms, but largely remain to be elucidated, including for coral. The ability of corals to acclimate and/or adapt to more extreme conditions^77^, potentially through immune memory, and/or immune training^79^, would clearly benefit restoration, conservation and management efforts. In this vein, genes underpinning traits of coral climate resilience are being sought as targets for assisted evolution, with a focus on cellular stress response components^10^. However, with our limited comprehension of holobiont immune mechanisms—those of the coral host, algal mutualists and the wider microbiome and their interactions—accurately measuring and interpreting factors that promote tolerance is challenging. In this context it seems we have a fair way to go before effective target genes will be identifiable.

The coral research community has high expectations of the more tolerant ‘super corals’ emerging^77,80^, being sought and being created^53,79^. But, whether evolved (assisted or otherwise) or physiological, enhanced tolerance (i.e., an increased damage threshold) will inevitably incur costs^24^. As well as ensuring coral reef persistence through increasing perturbations, such super corals will need to grow complex reef structures and reproduce sufficiently to maintain ecosystem function, while existing within unavoidable limits of life history trade-offs.

## Future directions and concluding remarks

Addressing the dramatic global loss of coral reefs is a daunting and bleak task. However, comprehensively approaching coral health under climate change from an immunological perspective will provide greater insight into survival mechanisms. In research efforts towards unraveling components of coral climate resilience it is imperative we use coral holobionts across the susceptibility range. This will avoid skewed, misleading and confounding results, which likely cannot be accurately generalized to other holobionts or locations. We also need awareness that altering one component of the holobiont, such as a *Symbiodinium* strain or a single gene, will have knock-on effects that we have not begun to explore. To help conceptualize the dynamics of coral holobiont immunity I have presented a simplified model in the hope it can be used to more accurately interpret immune activity of the multipartite organism and firmly build our foundation of knowledge. With awareness that coral holobiont damage thresholds will likely be species- and system-specific, I hope this model can be used as a framework to develop more targeted approaches and more effective monitoring of reef management, conservation and restoration, including genetic engineering.

Dramatically reducing carbon emissions is key to globally conserving coral reefs. While this largely hinges on political agenda, biologists can utilize a combination of immunological information and physiological natural history to better understand drivers of coral health. In doing so, and while pushing for action on climate change, hopefully we can buy enough time to conserve sufficient coral to maintain ecological function.
